# Body Mass Index, Muscle Strength and Physical Performance in Older Adults from Eight Cohort Studies: The HALCyon Programme

**DOI:** 10.1371/journal.pone.0056483

**Published:** 2013-02-20

**Authors:** Rebecca Hardy, Rachel Cooper, Avan Aihie Sayer, Yoav Ben-Shlomo, Cyrus Cooper, Ian J. Deary, Panayotes Demakakos, John Gallacher, Richard M. Martin, Geraldine McNeill, John M. Starr, Andrew Steptoe, Holly Syddall, Diana Kuh, on behalf of the HALCyon study team

**Affiliations:** 1 Medical Research Council Unit for Lifelong Health and Ageing and Institute of Epidemiology and Health Care, University College London, London, United Kingdom; 2 Medical Research Council Lifecourse Epidemiology Unit, University of Southampton, Southampton, United Kingdom; 3 School of Social and Community Medicine, University of Bristol, Bristol, United Kingdom; 4 National Institute for Health and Research Musculoskeletal Biomedical Research Unit, University of Oxford, Oxford, United Kingdom; 5 Centre for Cognitive Ageing and Cognitive Epidemiology, University of Edinburgh, Edinburgh, United Kingdom; 6 Department of Epidemiology and Public Health, University College London, London, United Kingdom; 7 Department of Primary Care and Public Health, Cardiff University, Cardiff, United Kingdom; 8 Institute of Applied Health Sciences, University of Aberdeen, Aberdeen, United Kingdom; 9 Geriatric Medicine Unit, Western General Hospital, Edinburgh, United Kingdom; University of Liverpool, United Kingdom

## Abstract

**Objective:**

To investigate the associations of body mass index (BMI) and grip strength with objective measures of physical performance (chair rise time, walking speed and balance) including an assessment of sex differences and non-linearity.

**Methods:**

Cross-sectional data from eight UK cohort studies (total N = 16 444) participating in the Healthy Ageing across the Life Course (HALCyon) research programme, ranging in age from 50 to 90+ years at the time of physical capability assessment, were used. Regression models were fitted within each study and meta-analysis methods used to pool regression coefficients across studies and to assess the extent of heterogeneity between studies.

**Results:**

Higher BMI was associated with poorer performance on chair rise (N = 10 773), walking speed (N = 9 761) and standing balance (N = 13 921) tests. Higher BMI was associated with stronger grip strength in men only. Stronger grip strength was associated with better performance on all tests with a tendency for the associations to be stronger in women than men; for example, walking speed was higher by 0.43 cm/s (0.14, 0.71) more per kg in women than men. Both BMI and grip strength remained independently related with performance after mutual adjustment, but there was no evidence of effect modification. Both BMI and grip strength exhibited non-linear relations with performance; those in the lowest fifth of grip strength and highest fifth of BMI having particularly poor performance. Findings were similar when waist circumference was examined in place of BMI.

**Conclusion:**

Older men and women with weak muscle strength and high BMI have considerably poorer performance than others and associations were observed even in the youngest cohort (age 53). Although causality cannot be inferred from observational cross-sectional studies, our findings suggest the likely benefit of early assessment and interventions to reduce fat mass and improve muscle strength in the prevention of future functional limitations.

## Introduction

Maintaining physical capability, defined as the ability to undertake the physical tasks of everyday living, is essential in older age. Lower levels of physical capability, as assessed by simple objective measures of physical performance (walking speed, chair rise and standing balance times) and muscle strength, have been shown to predict the onset of disability, loss of independence and survival in older community-dwelling populations [1]–[3]. It is therefore important to establish modifiable risk factors related to these measures.

The rise in the prevalence of obesity in all age groups in many countries [4], [5] coupled with the global ageing of the population means that establishing the influence of adiposity on physical capability is increasingly important from a public health perspective. Higher body mass index (BMI) has been associated with slower walking speed and poorer chair rise and standing balance performance [6]–[16], but studies are limited in a number of ways. Few have examined sex differences, most have focussed on either overweight/obesity or on a continuous measure of BMI assuming a linear relationship, and have not considered the influence of being underweight or investigated potential non-linearity and few have considered alternative measures of adiposity such as waist circumference [17]. The relationship between BMI and grip strength is less consistent [13], [18], but as weaker muscle strength has been associated with reduced levels of physical performance [17], there is a growing interest in whether sarcopenic obesity (a combination of weak muscle strength and high adiposity) [19] is particularly detrimental to physical performance [7], [19].

Healthy Ageing across the Life Course (HALCyon) is a collaborative research programme including nine UK cohorts (age range 50 years to 90+ years) that aims to investigate how factors across life influence physical capability and other aspects of healthy ageing. We investigate, in the eight cohorts with at least one objective measure of physical capability, the associations of BMI and, where available, grip strength with objective measures of physical performance (chair rise time, walking speed and standing balance). We also investigate the joint effects of BMI and muscle strength on physical performance, and test whether grip strength modifies the effect of adiposity. We assess whether there are sex differences in each of the main associations, and whether there is evidence of non-linearity. Finally, we consider whether waist circumference is associated with the outcome measures in a similar way to BMI.

## Methods

Data from the eight HALCyon cohorts [20] with relevant information were used in these analyses. Written informed consent was given by all participants as appropriate. Ethical approval was obtained from the Multicentre Research Ethics Committee for Scotland, the Ethics Committee of the Division of Medicine of the former South Glamorgan Area Health Authority and Gwent Research Ethics Committee, the Multicentre Research and Ethics Committee, the South East Multicentre Research Ethics Committee, the Bedfordshire and Hertfordshire Local Research Ethics Committee and the West Hertfordshire Local Research Ethics Committee, and the North Thames Multicentre Research Ethics Committee.

The Aberdeen Birth Cohort 1936 (ABC1936) includes men and women born in 1936 who sat a test of mental ability in 1947 as part of the Scottish Mental Survey [21]. A total of 70,805 children sat the test. In the 1990s those still resident in the Grampian area were identified through record linkage with lists of those registered with a General Practitioner. The first wave of new data was collected when study members were aged 62–68 years when 508 participated. Of these 498 (98.0%) contributed to analyses.

The Boyd Orr study is taken from an original sample of 4999 men and women born between 1918 and 1939 who participated in the Carnegie (Boyd Orr) Survey of Diet and Health in Pre-War Britain, 1937–1939 [22]. A total of 3182 who were traced, still alive and resident in Britain in 1997–1998, were sent a questionnaire and 1648 (51.8%) responded. When study members were aged 63–83 years, a sub-sample of 405 (55.3%) of a target sample of 732 surviving study members living around four of the original survey centres underwent clinical examination, including assessments of physical performance. All 405 contributed to analyses.

The Caerphilly Prospective Study (CaPs) recruited 2512 men born between 1920 and 1939 when they were aged 45–59 years from the town of Caerphilly, South Wales and the adjacent villages [23]. For the second examination, the original cohort was supplemented with 447 men of a similar age who had moved into the study area. However, 561 men were lost from the cohort giving a total of 2398 men who participated in this second phase. Physical capability was measured in wave 5 when cohort members were aged 65–84 when 1195 (49.8% of those seen at second phase) attended the clinic, with 1145 (95.8% of those attending clinic) being included in analyses.

The English Longitudinal Study of Ageing (ELSA) was drawn from men and women born in the first half of the twentieth century, whose household participated in the Health Survey for England in 1998, 1999 and 2001. All households with one or more resident born before 1 March 1952 that participated in these three years of the Health Survey for England and gave permission to be re-contacted in future, were eligible for ELSA [24]. The individual response rate for the baseline ELSA interview, which took place in 2002–03, was 64.7%. Of the total 12,099 respondents, 11,391 were core members. Physical performance measures were recorded at wave 2 in 2004–2005 when 8780 core members (77.1% of those seen at baseline) participated. Of these 7225 (82.3%) provided all the information required to be included in analyses.

The Hertfordshire Ageing Study (HAS) is a cohort of men and women born in North Hertfordshire between 1920 and 1930 whose birth and infant records were available [25]. Of the 6803 live single births, a total of 1428 were traced, alive and living in North Hertfordshire at the time of the first follow-up. When aged 63–73 years, 717 (50.2% of target sample) attended a clinic for examination including grip strength of whom 714 (99.6%) are included in analyses with grip strength as an outcome. Performance tests were carried out at the second wave when 294 of the 717 who attended clinic at the first follow-up were seen in clinic again, and 290 (98.6%) were included in analyses.

In 1998–2004, men and women born in Hertfordshire between 1931 and 1939 and still living in the county were recruited to a larger study; the Hertfordshire Cohort Study (HCS). Of the 39,764 live births, 7106 were traced as still alive in Hertfordshire and registered with a General Practitioner (GP) in 1998 [26]. Permission to contact 6099 was obtained from GPs and of these 2997 (49.1%) attended a clinic examination at the first new wave of data collection when participants were aged 59–73 years. A total of 2983 (99.5%) were included in analyses.

The Lothian Birth Cohort 1921 (LBC1921) consists of men and women born in 1921, who sat a test of mental ability in 1932 as part of the Scottish Mental Survey. A total of 87, 498 children sat the test. In the 1990s, those still resident in the Lothian area were identified using lists of individuals registered with a general practitioner. Of the 1120 potential participants identified, 728 responses were received, of which 501 were eligible. Media advertisements identified another 368 eligible participants. In total, 550 (63.3% of those identified as eligible) joined LBC1921 and completed the first wave of data collection which took place when participants were aged 77–80 years [21]. Of these 544 (98.9%) were included in analyses.

The MRC National Survey of Health and Development (NSHD) is a sample of all the births (n = 5362) that took place in England, Scotland and Wales in one week in 1946 with prospective follow-up since birth [27]. At 53 years of age when physical performance was first measured, the target sample consisted of 3673 still alive and living in Britain. Contact was not attempted for those who had died (n = 469), emigrated (n = 461), had permanently refused to participate in the study (n = 640) or were living abroad at the time of interview (n = 119). Of the 3673, 2989 (81.4% of the target, 55.7% of original sample) were interviewed and examined in their own homes and 2930 (98.0%) were included in analyses.

### Physical Capability

Grip strength and walking speed have been measured in five cohorts, get up and go and chair rise time in four and balance in seven. Harmonisation of the physical capability measures across cohorts has been discussed in detail elsewhere [20].

Dynamometers were used to measure grip strength in all studies. The maximum recorded value of grip strength from multiple attempts was used in analysis.

Chair rising ability was measured as the time taken to rise from a sitting to a standing position and then sit down again five complete times in HAS, HCS and ELSA, and ten times in NSHD. We regressed the time taken for 5 chair rises on the time for 10 chair rises for younger ELSA participants and used the coefficients from regression equations to obtain predicted times for 5 chair rises in NSHD. As the distribution for chair rise time was skewed, natural logarithms of the times were taken, and then multiplied by 100 so regression coefficients could be interpreted as percentage changes [28]. For display purposes, in order that a higher value represented better performance we used –100×ln(chair rise time) in analyses. Regression coefficients can then be interpreted as the percentage decrease in chair rise time (i.e. better performance) per unit increase in the predictor variable [28].

In LBC1921 the time it took participants to walk as quickly as possible over a distance of 6 m was recorded. In all other cohorts, participants were timed walking at their normal pace over distances ranging from 3 m to 6 m. Walking times were converted to speeds (cm/s) to account for the different distances walked. A timed get up and go (TUG) test which recorded the time taken to get up from a chair, walk 3 m at a normal pace, turn around, return to the chair and sit back down was carried out in four studies (HAS, HCS, CaPs, BO). We included TUG speed (cm/s) for CaPs and BO in walking speed analyses.

Standing balance was assessed as the time, up to a maximum of 30 seconds that a one-legged stance could be maintained with eyes open in HAS, HCS, CaPs, BO, and NSHD. In ELSA, only participants aged 69 and under who completed all three stages of a series of tandem stands were asked to balance on one leg. Participants over 70 completed only the series of tandem stands. In ABC1936 whether or not participants were able to balance on one leg with their eyes open for 5 seconds was recorded. A binary variable indicating whether an individual was unable to balance for up to 5 seconds was created.

### Body Size

All cohorts measured height and weight according to study protocol at the same data collection wave as the measures of physical capability. BMI was calculated as weight(kg)/height(m)^2^. Six cohorts measured waist circumference.

### Statistical Analysis

Measures of physical capability and adiposity used in these analyses were generally taken from the first wave where they had been recorded concurrently (see Cooper et al [20] for details). For each set of analyses, equivalent multiple regression models (logistic regression for standing balance) were first fitted within each study. The random effects meta-analysis model [29] (selected *a priori* due to expected heterogeneity) was then used to obtain an overall estimate across all studies, and the percentage of variation between studies that cannot be attributed to within-study variation was examined using I^2^ [30]. Regression models were fitted to estimate the associations between BMI and grip strength and BMI and each of the three physical performance measures (adjusted for age and height) and between grip strength and each performance measure (adjusted for age and height) within each study, separately for men and women. Sex differences in effects were obtained (defined as the interaction between sex and BMI or sex and grip strength) in models including both sexes. To assess the linearity of associations, first quadratic terms were added to models and then BMI (or grip strength) was split into categories using quintiles. In all meta-analyses, age was considered as a potential source of heterogeneity by assessing how the study estimates varied by mean age of participants. In addition, meta-analyses of interactions between age and BMI obtained within each study were performed.

Finally, we assessed the relative importance of grip strength and BMI to physical performance in the five studies with relevant data (NSHD, ELSA, HCS, HAS, LBC1921). Models were fitted within each study including BMI and grip strength with adjustment for age and height. BMI by grip strength interaction terms were added to test whether the effect of adiposity was modified by grip strength. Similar models were repeated with waist circumference instead of BMI, as preliminary analyses including both adiposity measures in models resulted in a weakening of both effects.

All analyses for the continuous outcomes were repeated using standardised measures. As the overall conclusions were unaltered, these results are not presented, but it is highlighted when this standardisation resulted in reduced heterogeneity. All analyses were carried out in Stata version 10.

### Results

Summary characteristics of the cohorts are provided in Table 1. Mean BMI for both men and women was over 26 kg/m^2^ in all cohorts, thus many participants in the included cohorts were overweight.

**Table 1:** Characteristics of men and women in the 8 HALCyon cohorts.

	Mean (SD) or %							
	LBC1921	HAS	CaPs	Boyd Orr	HCS	ELSA	ABC1936	NSHD
**Men** (n^1^)	229	171	1145	182	1569	3259	241	1445
		411^2^			1086^3^			
Age (yr)	79.1 (0.59)	76.5 (2.29)	73.3 (4.17)	70.9 (4.38)	65.7 (2.89)	66.1 (9.31)	64.7 (0.93)	53 (0)
		67.5 (2.39) 2			67.6 (2.75) 3			
Grip strength (kg)	34.7 (7.37)	38.47 (8.08)	–	–	44.0 (7.51)	40.7 (9.56)	–	47.7 (12.20)
		38.26 (7.14)^2^						
Chair rise time	–	2.94 (0.30)	–	–	2.72 (0.21)	2.38 (0.32)	–	2.26 (0.29)
(logsec)					2.72 (0.22)^3^			
Walking/TUG speed	1.50 (0.40)	0.87 (0.18)	0.58 (0.13)	0.64 (0.14)	0.95 (0.14)	0.91 (0.27)	1.26 (0.25)	–
(m/s)					0.94 (0.15)^3^			
Unable to balance	–	31.0%	25.9%	18.8%	19.7%	10.3%	6.1%	3.5%
for 5 seconds					15.0%^3^			
BMI (kg/m^2^)	26.2 (3.53)	27.6 (4.05)	27.8 (3.99)	27.3 (3.82)	27.2 (3.76)	27.9 (4.29)	26.9 (3.52)	27.39 (4.01)
Height (cm)	171.4 (6.83)	171.4 (6.36)	170.1 (6.47)	171.7 (6.78)	174.1 (6.47)	172.7 (6.94)	171.9 (6.95)	174.7 (6.56)
**Women** (n^1^)	315	119	–	223	1414	3966	257	1485
		303^2^			1213^3^			
Age (yr)	79.1 (0.57)	76.5 (2.29)	–	70.6 (4.29)	66.6 (2.71)	66.6 (9.83)	64.7 (1.01)	53 (0)
		67.4 (2.23)^2^			67.6 (2.75)^3^			
Grip strength (kg)	20.6 (4.49)	23.64 (6.64)	–	–	26.52 (5.73)	24.3 (6.43)	–	27.7 (7.92)
		22.55 (5.29)^2^						
Chair rise time	–	3.03 (0.28)	–	–	2.88 (0.26)	2.42 (0.33)	–	2.28 (0.28)
(logsec)					2.86 (0.26)^3^			
Walking/TUG speed	1.31 (0.33)	0.78 (0.19)	–	0.64 (0.15)	0.93 (0.34)	0.84 (0.28)	1.20 (0.23)	–
(m/s)					0.92 (0.32)^3^			
Unable to balance	–	36.1%	–	21.1%	20.1%	16.6%	8.1%	5.3%
for 5 seconds					17.6%^3^			
BMI (kg/m^2^)	26.2 (4.57)	27.7 (5.13)	–	27.6 (4.87)	27.6 (4.91)	28.0 (5.33)	27.0 (4.92)	27.42 (5.41)
Height (cm)	157.5 (5.79)	158.3 (5.50)	–	158.0 (5.81)	160.8 (5.88)	159.2 (6.69)	159.0 (6.18)	161.6 (5.97)

1 Number of participants with a measure of BMI and at least one valid physical performance measure.

2 Grip strength measured at wave 1 (as well as wave 2). Performance tests measured only at wave 2.

3 Physical performance tests measured at either wave 1 or wave 2. LBC1921 = Lothian Birth Cohort 1921, CaPs = Caerphilly Prospective Study, HCS = Hertfordshire cohort study, HAS = Hertfordshire Ageing Study, ELSA = English Longitudinal Study of Ageing, ABC1936 = Aberdeen Birth Cohort 1936, NSHD = MRC National Survey of Health and Development.

### Adiposity and Grip Strength

After adjustment for age and height, higher BMI was associated with stronger grip strength among men only (Table 2, Figure S1) with heterogeneity across studies (I^2^ = 57.2%). There was a suggestion that associations were stronger at younger ages, but this variation was reduced when using standardised grip strength and no evidence of an interaction between age and BMI was found when pooling within-study terms. There was strong evidence of a sex difference in association when within-study differences (the sex by BMI interaction terms) were combined in a meta-analysis. Grip strength was 0.22 kg (95% CI: 0.17 to 0.28) greater for every kg/m^2^ higher BMI in men than women (Table 2). Men in the lowest fifth of BMI had a particularly low mean grip strength compared with men in the top four fifths (Figure S2). As only around a quarter of individuals were of normal weight or below (<25 kg/m^2^) these analyses investigated the associations with BMI primarily within the overweight and higher range. We therefore repeated analyses using the classification of underweight, normal weight, overweight (≥25 kg/m^2^) and obese (≥30 kg/m^2^). Underweight women, as well as underweight men, had weaker grip strength than those with higher BMI (Figure S3).

**Table 2:** Summary regression coefficients obtained by random effects meta-analysis of within-study estimates of the association between BMI and grip strength and BMI and the performance measures (models also include age, where appropriate, and height).

		Number of Studies	Number of Individuals	Overall regression coefficient per kg/m^2^ (95% CI)	I^2^	Sex by BMI interaction^1^ (women-men)	
						Overall regression coefficient per kg/m^2^(95% CI)	p-value
Grip strength (kg)	Men	5	6855	0.23 (0.14,0.31)	57.2%	−0.22 (−0.28, −0.17)	<0.001
	Women	5	7358	0.01 (−0.04,0.06)	54.0%		
Chair rise performance (%)	Men	4	4979	−0.76 (−1.07, −0.44)	50.4%	−0.32 (−0.65, 0.01)	0.06
	Women	4	5794	−1.04 (−1.19, −0.90)	0%		
Walking speed (cm/s)	Men	7	5085	−0.76 (−0.97, −0.56)	46.9%	−0.14 (−0.37,0.09)	0.2
	Women	6	4676	−0.92 (−1.19, −0.65)	57.3%		
Inability to balance	Men	7	6991	1.08 (1.04.1.11)	55.5%	1.03 (0.99,1.06)	0.1
for 5 seconds (OR)	Women	6	6930	1.11 (1.08,1.14)	55.9%		

1 Obtained from a meta-analysis of within-study interaction terms from models also including the main effects of BMI and sex (men coded as 0 and women coded as 1), and adjusted for age and height.

Higher waist circumference was related to stronger grip strength in men, but more weakly than BMI (data not shown). Including both BMI and waist circumference in the same model, resulted in the association with waist circumference becoming highly negative for both sexes, particularly for men. The positive association with BMI strengthened.

### BMI and Physical Performance

Higher BMI was associated with poorer chair rise performance, slower walking speed and greater odds of being unable to balance for 5 seconds in both sexes, after adjustment for age and height (Table 2, Figures S4, S5, S6). For chair rise performance, there was moderate heterogeneity among men with a trend suggesting weaker associations with decreasing mean cohort age. However, no evidence of an age by BMI interaction was found when combining within-study estimates (p = 0.2). Exclusion from the walking speed analysis of the two studies (BO and CaPs) with TUG speed resulted in little change to the results. Heterogeneity in the associations among women with walking speed was explained by LBC1921 and heterogeneity among men was reduced to zero when a standardised outcome was used. The oldest cohort, HAS, was responsible for much of the heterogeneity among estimates for standing balance in both sexes (I^2^ reduced to 0 for men and 27.6% for women after exclusion). There was some suggestion that the associations were stronger in women than men, although these differences were small (Table 2).

**Figure 1:**
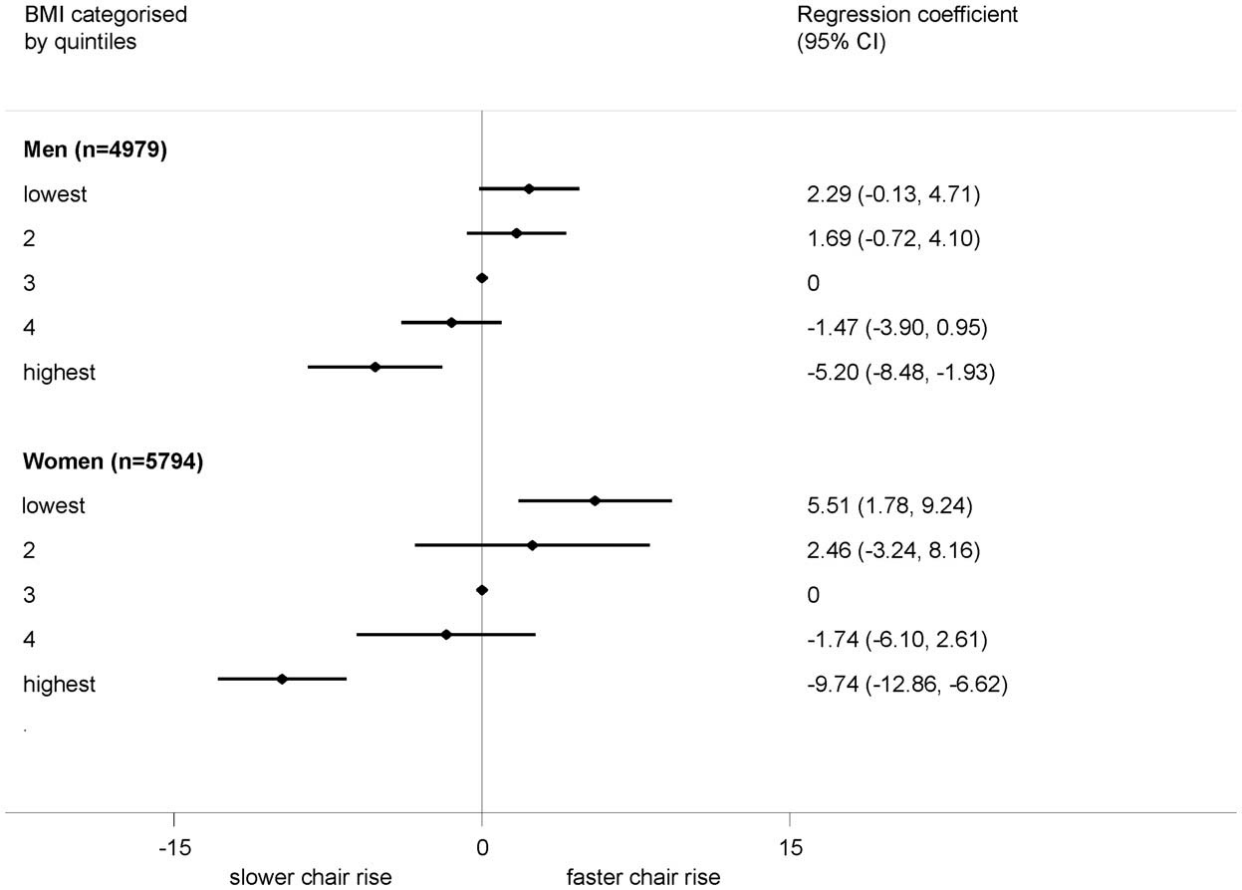
Association between BMI (categorised into fifths) and chair rise performance (%). Footnote: Summary estimates (each category compared with the middle category) from a random effects meta-analysis (4 studies) are presented. Models adjusted for age (where appropriate) and height.

**Figure 2:**
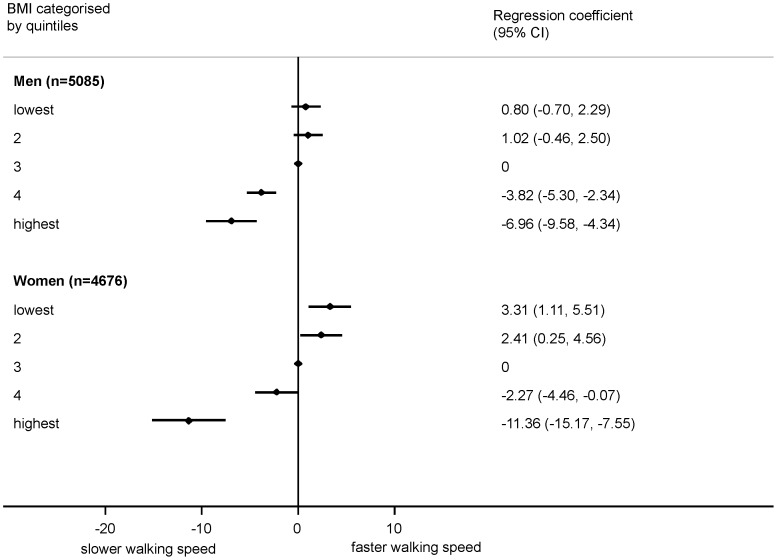
Association between BMI (categorised into fifths) and walking speed (cm/s). Footnote: Summary estimates (each category compared with the middle category) from a random effects meta-analysis (7 studies for men, 6 studies for women) are presented. Models adjusted for age (where appropriate) and height.

**Figure 3:**
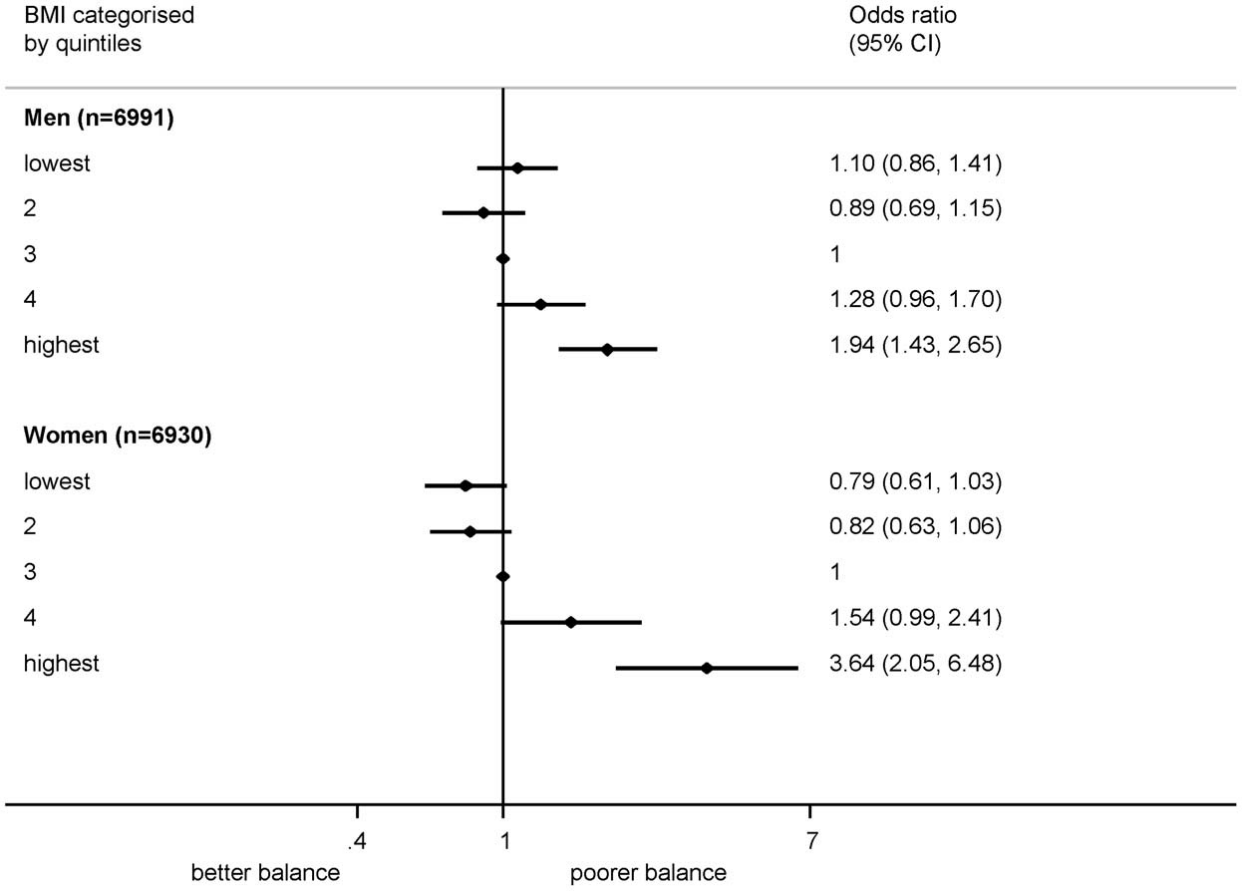
Association between BMI (categorised into fifths) and inability to balance for 5 seconds (OR). Footnote: Summary estimates (each category compared with the middle category) from a random effects meta-analysis (7 studies for men, 6 studies for women) are presented. Models adjusted for age (where appropriate) and height.

When quadratic terms were pooled in a meta-analysis, a non-linear effect of BMI on walking speed was suggested in both sexes (p<0.001 in both) and on standing balance among men (p = 0.001). There was less evidence of a consistent deviation from linearity for chair rise time. When considering BMI in categories, for all three measures, but especially for walking speed and standing balance, the detrimental impact of BMI was particularly evident in the highest fifth of the BMI distribution (Figures 1,2, 3). Using the standard categorisation of underweight, normal weight, overweight and obese, for chair rise performance, there was little difference between the underweight group and the normal weight group, with only the obese group exhibiting substantially poorer performance (Figure S7). For both walking speed and standing balance, the underweight group showed poorer performance than the normal weight group (Figures S8, S9). However, the confidence intervals were wide due to the small numbers of underweight individuals.

### Grip Strength and Physical Performance

**Figure 4:**
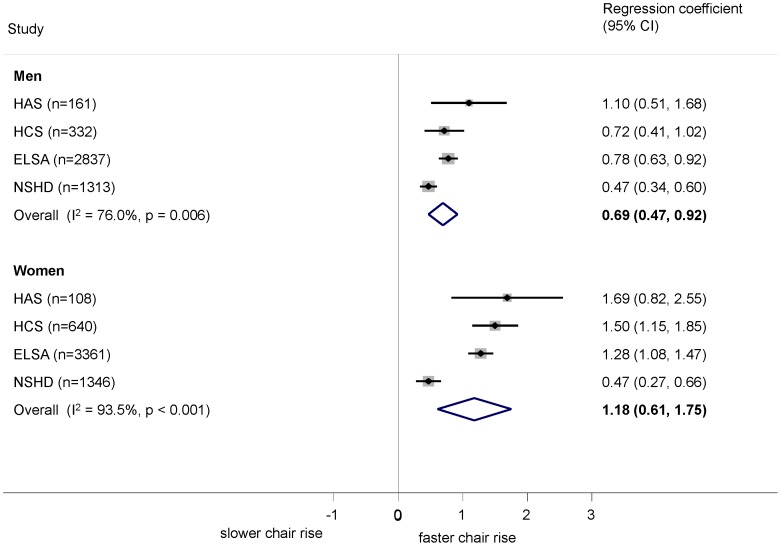
Association between grip strength (kg) and chair rise performance (%). Footnote: Models adjusted for age (where appropriate) and height.

**Figure 5:**
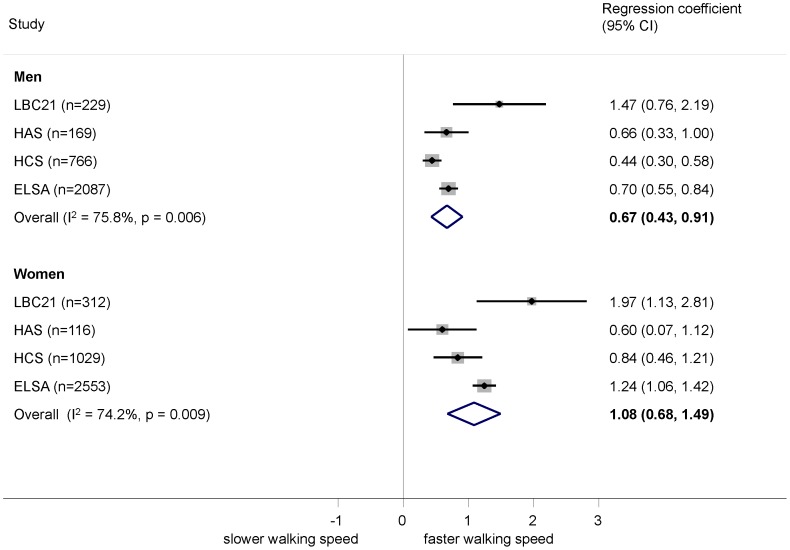
Association between grip strength (kg) and walking speed (cm/s). Footnote: Models adjusted for age (where appropriate) and height.

**Figure 6:**
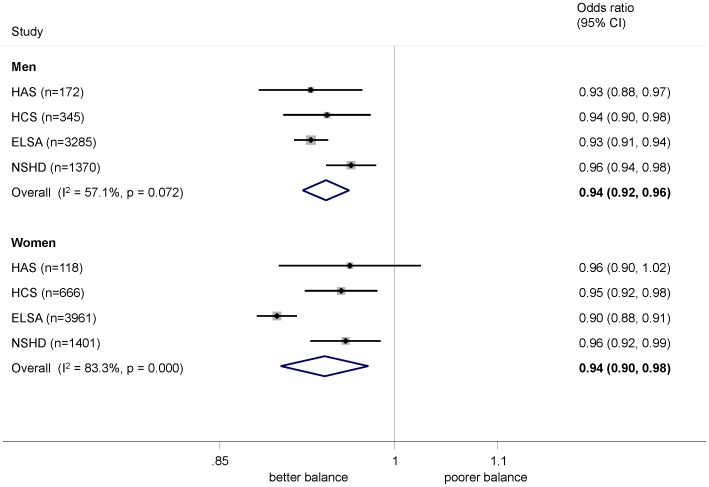
Association between grip strength (kg) and inability to balance for 5 seconds (OR). Footnote: Models adjusted for age (where appropriate) and height.

Higher grip strength was associated with better performance on all tests in both men and women after adjustment for age and height (Figures 4, 5, 6). The relationship was stronger in women than men for chair rise performance (0.36% (−0.03, 0.75) per kg greater grip strength, p = 0.07) and walking speed (0.43 cm/s (0.14, 0.71), p = 0.004). The sex difference in chair rise performance became considerably stronger (p<0.001) on excluding the youngest cohort, NSHD, which was also the source of heterogeneity. There was no evidence of a sex difference in the relationship between grip strength and standing balance (OR (95% CI) for interaction: 0.99 (0.95, 1.02), p = 0.5). Although there was a suggestion (Figures 4 and 5) that the effects were stronger at older ages for chair rise performance and walking speed, this was not supported when combining the within study grip strength by age interaction terms (p>0.2 in all cases).

A non-linear effect of grip strength on chair rise performance and walking speed was observed in both sexes, with the additional beneficial effects of stronger grip strength becoming less at higher levels of strength. There was no evidence of non-linearity (on the log scale) for standing balance.

### Combined Effects of BMI and Grip Strength on Physical Performance

Both grip strength and BMI remained associated with chair rise time, walking speed and standing balance in mutually adjusted models (also adjusted for age and height) (Table 3). There was no evidence of an interaction between BMI and grip strength for any of the performance measures. Given the previously observed non-linearity of relationships for both BMI and grip strength, further models using binary categorisations of BMI (highest fifth (>30.5 kg/m^2^ in men and BMI>31.7 kg/m^2^ in women) versus rest) and grip strength (lowest fifth (<32 kg in men and <18 kg in women) versus rest) were fitted (Table 3). Both BMI and grip strength showed strong independent effects, but, again, there was no evidence of an interaction for any performance outcome. Hence, an additive effect is suggested.

**Table 3:** Summary regression coefficients obtained from random effects meta-analysis of within-study estimates from models including both BMI and grip strength (models also include age, where appropriate, and height) in four studies.

			Grip and BMI as continuous variables in same model				Grip and BMI as binary variables^1^ in same model			
		N	Grip strength	I^2^	BMI	I^2^	Grip strength	I^2^	BMI	I^2^
			regression coefficient per kg (95% CI)		regression coefficient per kg/m^2^ (95% CI)		regression coefficient kg (lowest fifth v rest) (95% CI)		regression coefficient kg/m^2^ (highest fifth v rest) (95% CI)	
Chair rise	Men	4614	0.74 (0.49,1.00)	81%	−0.81 (−10.8, −0.54)	28%	−10.78 (−12.98, −8.59)	0%	−5.50 (−8.55, −2.45)	40%
performance (%)	Women	5407	1.21 (0.60,1.81)	95%	−1.14 (−1.28, −0.99)	0%	−13.45 (−19.23, −7.70)	83%	−10.62 (−12.54, −8.71)	0%
Walking	Men	3222	0.69 (0.44,0.94)	79%	−1.03 (−1.41, −0.64)	69%	−10.97 (−13.48, −8.46)	21%	−8.54 (−12.84, −4.23)	74%
speed (cm/s)	Women	3972	1.11 (0.69,1.53)	77%	−1.15 (−1.57, −0.73)	72%	−13.52 (−15.60, −11.45)	0%	−12.90 (−18.34, −7.43)	70%
Balance (OR)	Men	5128	0.94 (0.92,0.96)	60%	1.10 (1.04,1.17)	71%	2.64 (2.09,3.32)	0%	2.36 (1.62,3.46)	46%
	Women	6074	0.94 (0.90,0.98)	83%	1.12 (1.08,1.15)	64%	2.65 (2.21,3.17)	0%	2.98 (2.01,4.42)	63%

1 Binary variable for grip strength is defined as the lowest fifth versus the highest four fifths and binary variable for BMI is defined as the highest fifth versus the lowest four fifths. Hence regression coefficients display the difference for the at risk group versus the rest for both variables.

Findings in the three studies with all relevant measures at the same age were very similar when waist circumference replaced BMI in these analyses.

## Discussion

Higher BMI was associated with poorer performance on chair rise, walking speed and standing balance tests. The associations of BMI with performance were non-linear, with poorer performance primarily observed in the most overweight groups, but with some suggestion of poorer performance also in the underweight. Weaker grip strength was associated with poorer performance on all tests and the associations with some aspects of performance were stronger in women than men. Although higher BMI was correlated with higher grip strength in men, BMI remained independently associated with performance after adjustment for grip strength. Those in the highest fifth of BMI and the lowest fifth of grip strength had the poorest performance through an additive effect. The associations with waist circumference were similar to those for BMI.

### Strengths and Limitations

A major strength of this study is the large sample size obtained by combining data from eight cohorts. This results in adequate power to investigate sex differences and to examine in detail the shape of relationships. The harmonisation of data and the coordinated analyses allows for an assessment of consistency of findings across studies, thus making conclusions more robust. Another strength is the use of objective measures of physical capability that have high levels of reliability and which allow examination of variation in function across the full spectrum of ability. Although data were harmonised, there remain differences in measures across studies which might limit comparability. Standing balance performance was particularly problematic to harmonise; the measure of balance selected is less appropriate for younger studies such as NSHD as very few individuals of that age were unable to balance for 5 seconds [20]. We did conduct a number of sensitivity analyses (for example excluding studies using TUG speed instead of walking speed) and found little differences in associations.

All associations considered were cross-sectional due to the lack of comparable multiple measurements of BMI and physical performance across studies, thus limiting our ability to deduce the direction of association. Hence, it is possible that the observed associations are actually due to reductions in physical capability resulting in increases in BMI, possibly through reduced physical activity. Previous work in the NSHD does, however, suggest that prior life course body size impacts on physical performance at age 53 [31]. The study designs of the HALCyon cohorts vary, with the samples analysed here being obtained in different ways and none remain completely representative of the original populations from which they were selected. Hence, findings could have been influenced by sample selection and selective attrition. However, although all studies are from the UK, given their diverse designs and selection criteria any such bias would be unlikely to be completely consistent across all studies. That we observed consistent findings in terms of direction of associations, if not magnitude, suggests that the results are not entirely due to selection bias within cohorts. There is also the possibility that the studies included in HALCyon may not be representative of all studies which could address the aims of this research. The extent of heterogeneity (I^2^) could not be estimated very precisely as the number of studies included was relatively small. This also limited the extent to which we could investigate reasons for heterogeneity. *A priori* it was considered that age may have an impact on the strength of associations. As well as ordering plots by mean age of study, we tested whether there was evidence of a BMI by age interaction. In most studies the age range may have been too narrow to properly assess this.

### Comparison with Previous Studies

Other studies have shown similar associations between higher BMI and poorer physical capability [7]–[16]. Consistent with previous findings of a stronger association between BMI and functional limitations in women than men [6], [32], [33], we find weak evidence to support a small sex difference in relation to physical performance, particularly chair rising.

For walking speed and standing balance, the underweight group appeared to perform more poorly than the normal and overweight groups, although the small proportion (3%) underweight (even when defined as <20 rather than <18.5 kg/m^2^) meant we could not test the difference adequately. The finding is in agreement with some previous studies [12], [13]. We also found only small mean differences in physical performance with higher BMI within the normal to overweight categories with greater differences only occurring in the top one or two fifths of the BMI distribution. As in the few previous studies, we also found that higher central adiposity was related to slower walking speed [17], and this was extended to other measures of performance. Rather than adding additional information, however, we found that waist circumference acted in a very similar way to BMI in relation to performance.

Previous studies have generally investigated the combined effects of grip strength and adiposity by defining four groups: neither sarcopenic (as measured by low grip strength or muscle mass) nor obese; obese only; sarcopenic only; and both obese and sarcopenic. Some, but not all, have found that poor performance is greatest in those with both obesity and sarcopenia. This may depend on the way that sarcopenia has been defined, with those basing it on low muscle strength showing an effect [8], [34], but those with muscle mass not [35], [36]. Studies based on grip strength were consistent with our findings of poorest performance among those with low strength and high BMI, with the effects of the two components being additive. Given the methods used in previous studies it is generally unclear whether the estimated effects of sarcopenic obesity simply reflect an additive effect of low muscle strength and obesity or whether the detrimental effect of obesity is only apparent if accompanied by low muscle strength (i.e. an interaction).

### Explanations and Implications

The curvilinear relationships observed between BMI and chair rise and standing balance times suggest that there may be a threshold for BMI which is detrimental to these outcomes. This may support categorisation of BMI in analyses, although a threshold effect, previously suggested from a review (but not meta-analysis) of the literature [6] as being between 30–35 kg/m^2^, was not clearly observed in our study across all cohorts and performance measures, suggesting the focus should not just be on the extreme category.

Poor health, low levels of physical activity and frailty may explain the finding that underweight participants performed more poorly than normal and overweight individuals. However, the cross-sectional nature of these analyses means that the direction of any relationships cannot be determined. It may also be that any such relationship is weakened by those not able to perform the tests being in worse health, and thus perhaps more likely to be underweight than those that are able. Associations between weak grip strength and high adiposity and poor performance may also be in part due to the ill health and low levels of physical activity in these groups [19].

Stronger effects of BMI on performance among women compared with men may reflect differences in body composition between the sexes. Due to genetic, hormonal and environmental differences women tend to have a lower proportion of lean mass than males. There are also gender differences in the distribution of lean mass with males tending to have greater amounts of upper body lean mass [37]. This is supported by the positive association between BMI and grip strength in men but not women; although even among men, it was only those in the lowest BMI group who exhibited lower muscle strength compared with the others. Grip strength was also generally more strongly associated with performance in women than men, perhaps because women have much lower strength than men with more, therefore, being at risk of impairment.

We found substantial heterogeneity in associations across studies for some analyses. In some cases, such as for walking speed, this was reduced on use of a standardised outcome due to differences in the standard deviations across studies resulting from variations in protocol. For chair rise time and standing balance the suggestion that the association with BMI got stronger with increasing mean age of study participants may be due to the same BMI representing a greater proportion of fat mass at older ages as fat mass has been shown to increase with age while muscle mass declines [38], [39]. However, any such age-related change in the effect of body size should be interpreted with caution as our observations were at the study, rather than individual, level, and analyses within studies found no evidence of a change in effect with age. Heterogeneity might also exist due to the different life course experiences of the different cohorts. Cross-sectional associations between BMI and physical performance are likely to depend not only on current size but also on the length of time that an individual has been overweight. Different cohorts have experienced rises in mean BMI at different ages and thus for the same BMI, the burden of cumulative BMI may be different [38], [40]. Alternatively, differences may be a result of variation in study design and conduct.

Those at the bottom end of the grip strength distribution, in general, did particularly poorly on all performance tests suggestive, as for BMI, of a threshold effect. Hence, individuals with poor muscle strength and high adiposity (sarcopenic obesity) have considerably poorer performance than others through an additive effect. These associations with physical performance were evident even in the youngest cohort (53 years). Although we cannot infer causality from our findings, they suggest that early assessment to identify those most at risk, and interventions to reduce fat mass and improve muscle strength, may prevent future functional limitations.

## Supporting Information

Figure S1 **Association between BMI (kg/m^2^) and grip strength.**
(TIF)

Figure S2 **Association between BMI (categorised into fifths) and grip strength (kg).** Summary estimates (each category compared with the middle category) from a random effects meta-analysis (5 studies). Models adjusted for age (where appropriate) and height.
(TIF)

Figure S3 **Association between BMI (in categories) and grip strength (kg).** Summary estimates (each category compared with the normal weight category) from a random effects meta-analysis (5 studies). Models adjusted for age (where appropriate) and height.
(TIF)

Figure S4 **Association between BMI (kg/m^2^) and chair rise performance (%).**
(TIF)

Figure S5 **Association between BMI (kg/m^2^) and walking speed (cm/s).**
(TIF)

Figure S6 **Association between BMI (kg/m^2^) and inability to stand on one leg for 5 seconds (OR).**
(TIF)

Figure S7 **Association between BMI (in categories) and chair rise performance (%).** Summary estimates (each category compared with the normal weight category) from a random effects meta-analysis (4 studies). Models adjusted for age (where appropriate) and height.
(TIF)

Figure S8 **Association between BMI (in categories) and walking speed (cm/s).** Summary estimates (each category compared with the normal weight category) from a random effects meta-analysis (7 studies for men, 6 studies for women). Models adjusted for age (where appropriate) and height.
(TIF)

Figure S9 **Association between BMI (in categories) and inability to balance on one leg for 5 seconds.** Summary estimates (each category compared with the normal weight category) from a random effects meta-analysis (5 studies for men: HAS and ABC1936 are omitted due to small numbers, 4 studies for women: NSHD and ABC1936 are omitted due to small numbers). Models adjusted for age (where appropriate) and height.
(TIF)
