# Supplementary figures and images for: Body Mass Index, Muscle Strength and Physical Performance in Older Adults from Eight Cohort Studies: The HALCyon Programme
Source: PLoS One. 2013 Feb 20;8(2):e56483. doi: 10.1371/journal.pone.0056483 (PMC3577921; doi:10.1371/journal.pone.0056483)

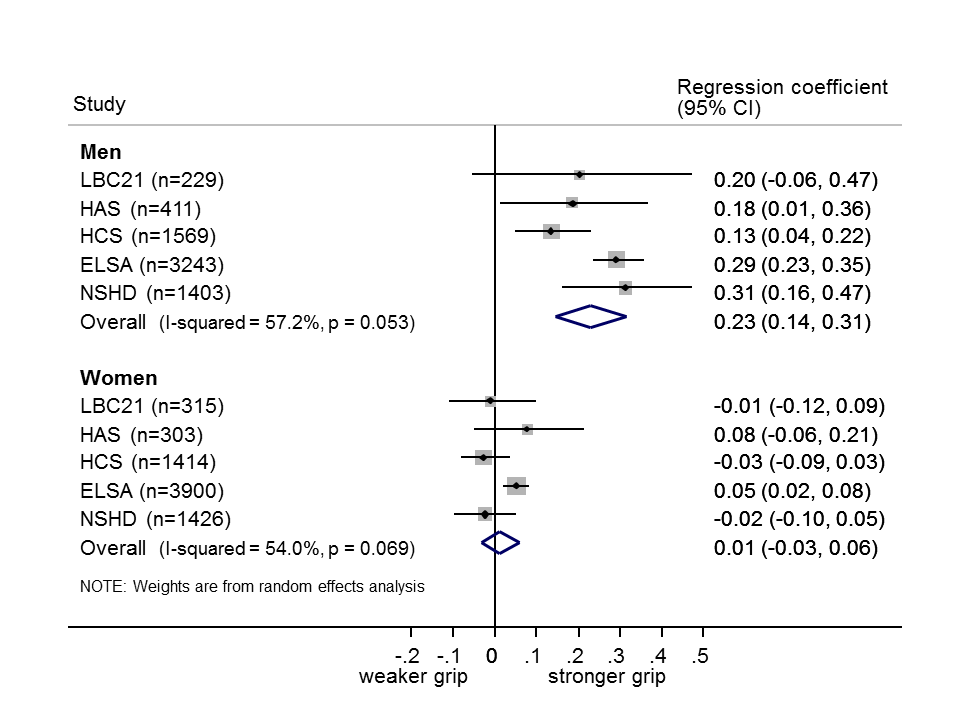

Supplement: Figure S1 — Association between BMI (kg/m2) and grip strength. (TIF) [file pone.0056483.s001.tif]

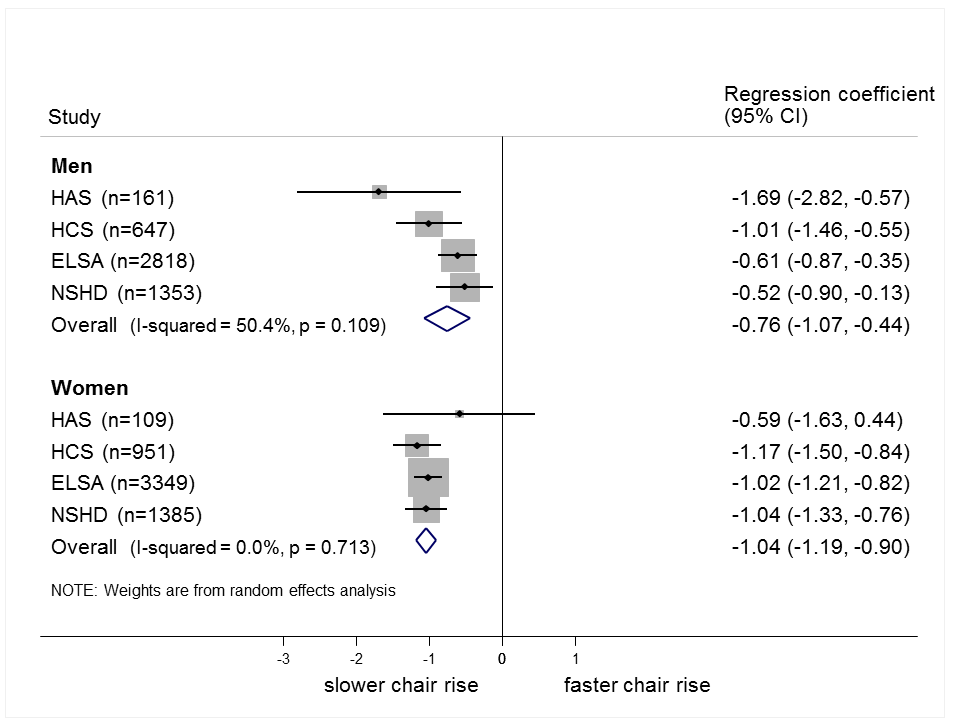

Supplement: Figure S4 — Association between BMI (kg/m2) and chair rise performance (%). (TIF) [file pone.0056483.s004.tif]

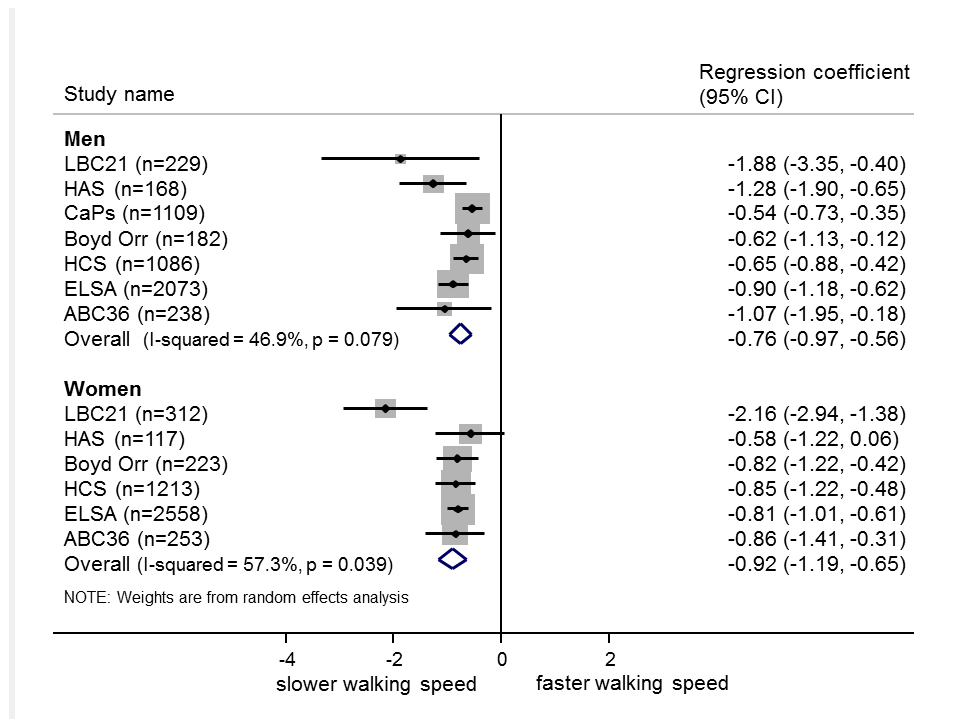

Supplement: Figure S5 — Association between BMI (kg/m2) and walking speed (cm/s). (TIF) [file pone.0056483.s005.tif]

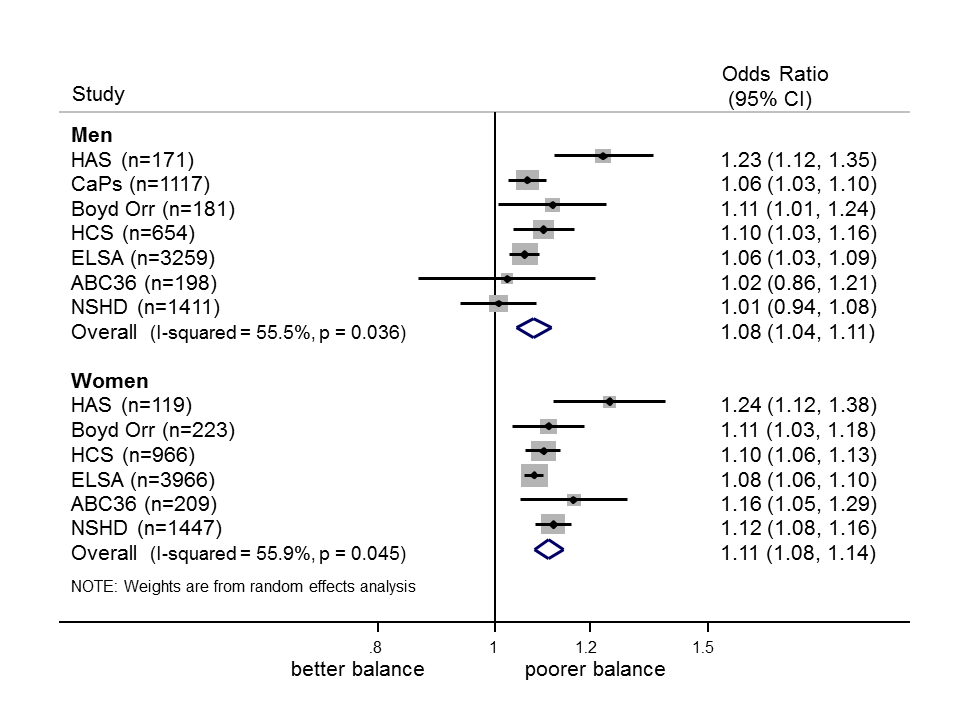

Supplement: Figure S6 — Association between BMI (kg/m2) and inability to stand on one leg for 5 seconds (OR). (TIF) [file pone.0056483.s006.tif]
